# Functional and oncologic outcomes after nephron-sparing surgery in a solitary kidney: 10 years of experience

**DOI:** 10.1590/S1677-5538.IBJU.2014.0463

**Published:** 2016

**Authors:** José Ignacio Costabel, Patricio García Marchiñena, Federico Tirapegui, Augusto Dantur, Alberto Jurado, Guillermo Gueglio

**Affiliations:** 1 Departamento de Urología del Hospital Italiano de Buenos Aires, Argentina

**Keywords:** Renal carcinoma, solitary kidney, nephron-sparing surgery, partial nephrectomy

## Abstract

**Objectives:**

To evaluate functional and oncologic outcomes of partial nephrectomy (PN) in patients with a solitary kidney.

**Materials and Methods:**

A retrospective analysis of patients with a solitary kidney undergoing nephron-sparing surgery between March 2003 and March 2013 was performed. GFR was recorded before the procedure and 3 months after surgery, thus establishing a change (cGFR). Several variables that may influence cGFR were analyzed. Complications are herein described, namely bleeding, fistula, acute renal failure and end-stage renal disease (ESRD). Local recurrence and margin status are also described. Survival rates were calculated using the Kaplan Meier method (2 patients with metastasis at the time of surgery were excluded from the analysis).

**Results:**

Forty-five patients were available for analysis. Median follow-up was 27.56 months (r 3-96). Mean cGFR was-7.12mL/min (SD 2.1). Variables significantly related with lower GFR after surgery were loss of renal mass (p=0.01)) and male gender (p=0.03). Four patients (8.8%) experienced hemorrhage. Nine patients (20%) developed a urinary fistula. Only one patient with bleeding required open surgery. Two patients (4.4%) needed transient dialysis. Three patients (6.6%) developed ESRD. Four patients (8.8%) had positive surgical margins (PSMs) and four patients (88%) had local recurrence (2 of these had PSMs). Five patients (11.1%) died during follow-up. Four patients (8.8%) died because of renal cancer. Estimated 2-year overall survival, disease-free survival and cancer specific survival rates were 88.4% (CI 95% 70.5-96); 87.7% (CI 95% 68.1-96) and 92.4% (CI 95% 75-98), respectively.

**Conclusion:**

Loss of renal mass and male gender were associated with lower postoperative GFR. Our outcomes were comparable with those in the World literature.

## INTRODUCTION

While nephron-sparing surgery is ideally used for renal masses in patients with a solitary kidney (1), it is not always possible, rendering a patient anephric and the need of renal replacement therapy. This situation presents a challenging scenario for the urologist since cancer control, with the maximum preservation of functioning parenchyma, is mandatory.

Since the risk of a synchronous contralateral renal tumor does not decrease significantly with time (2), it is likely that the number of patients with a tumor in a solitary kidney will increase. Given the lack of a normally compensating contralateral kidney, this patient population represents an “ideal” model to study the functional aspects of the kidney undergoing partial nephrectomy.

The aim of this study is to evaluate the functional outcomes of partial nephrectomy in patients with a solitary kidney at our institution. Clinical and pathological variables were analyzed in order to determine the association with lower glomerular filtration rates (GFR) in the late postoperative period. Oncologic outcomes are also herein described.

## MATERIALS AND METHODS

### Patient selection and characteristics

Partial nephrectomies in patients with a solitary kidney performed at our institution from March 2003 to March 2013 were included in the study. Patients with prior renal surgery in the solitary kidney (n=2), patients with lesions on a transplanted kidney (n=2) and patients who had undergone bench surgery and auto-transplantation (n=3) were excluded. Data from patients was prospectively collected in a standardized database except for the data from 16 patients who had undergone surgery before this database was opened (2010). In these patients, data was collected from the electronic medical record. A retrospective analysis of this data therefore was performed.

Age, gender, side of surgery, symptoms (pain or hematuria), single anatomical/functional kidney (contralateral atrophy) along with comorbidities are herein described. Data from the pathology report (histological type and multifocality) is also included.

### Surgical technique

Each procedure was performed by the same two urologists responsible for the Retroperitoneal Surgery Section (G.G and A.J). Surgery was planned based on CT scan with I.V contrast to determine vascular and tumor condition. MRI was requested when the creatinine serum level was higher than 1.6mg/dL. The use of arterial clamping was decided based on preoperative images but, ultimately, on intraoperative findings.

Flank incision was routinely used for the open approach. Manual compression was the first option to avoid ischemia. When not possible, cold ischemia was applied.

For the laparoscopic approach, authors used a retroperitoneal access. Warm ischemia was used in all laparoscopic cases.

There were three possible surgical techniques based on tumor location: enucleation (resection of the tumor only); polar nephrectomy (resection of the entire pole including single or multiple lesions) or hemi-nephrectomy (half of the kidney was resected including single or multiple lesions). Enucleation was the first choice whenever possible (exophytic lesions without sinus involvement).

Intraoperative frozen section was routinely performed to assess negative margins. If frozen section was positive for cancer, resection was extended until negative margin was achieved. In addition, argon beam coagulation was routinely used.

A CT-1 needle with 3-zero polyglactin was used to perform meticulous running suture repair of the collecting system. Renorrhaphy was performed with trans-parenchymal 2.0 polyglactin suture. The parenchymal sutures were cinched down over 1 to 2 bolsters, thereby securely re-approximating the renal parenchyma and achieving hemostasis.

To assess the loss of renal mass, the maximum diameter (in millimeters) of the surgical specimen obtained was used. In case of multifocality, maximum diameters of single surgical pieces were added if the tumors were resected separately.

### Functional outcomes

GFR was estimated using the abbreviated MDRD equation (GFR in mL/min/1.73m2=186 x sCr1, 154 x age 0.203 x (0.742 if female) x (1.210 if black) (3).

For the evaluation of functional outcomes we calculated a change in GFR (cGFR) defined as the difference between the mean GFR before surgery and the mean GFR 3 months after surgery. We did not consider the immediate postoperative GFR because it is influenced by many factors (e.g. anesthetic drugs, analgesics, hydration, etc).

Regression models were used to examine whether any of the following variables were significantly associated with the cGFR:

– Age
– Gender
– Functional (contralateral renal atrophy) versus anatomical mono-renal
– Renal mass loss (as defined above)
– Multifocality
– Ischemia (yes/no)
– Ischemia time (in minutes)
– Body mass index (BMI)
– Coronary artery disease
– Arterial hypertension (AHT)

### Diabetes

Immediate postoperative complications were as follows: bleeding (diagnosed with clinical features and confirmed with red blood cell count in the drainage), fistula (defined as urine drainage higher than 50mL/day 72 hours after surgery (4) and transient dialysis. End-stage renal disease (ESRD) was defined as need for permanent dialysis.

### Oncologic outcomes

Surgical margins are described. The presence of tumor cells at the inked margin of the resection was considered to represent a positive surgical margin (PSM). Follow-up is described in months (from date of surgery). Local recurrence is described as a parameter of oncologic outcomes. The 2-year overall survival, disease-free survival (time to local recurrence) and cancer specific survival rates were estimated. Two patients with metastases at the time of surgery were excluded from this analysis.

### Statistical analysis

Continuous variables are summarized as mean and standard deviation (SD) in case of normal distribution (tested with Schapiro Wilk test); otherwise they are expressed as median and range. Categorical variables are reported as absolute values and percentages. Univariate and multivariate linear regression models were fit to assess which clinical or pathological features were associated with outcome. The multivariate model was constructed using a stepwise selection procedure with p≤0.05 needed for a variable to enter the model. The relationships of outcome with clinical and pathological features were summarized by linear regression model coefficients (β) and by the 95% confidence interval (CI). All statistical tests were assessed with a significance level of p≤0.05 or CI not including 0. The Kaplan Meier method was used to estimate survival rates. The software used was PASW18.0 R.

## RESULTS

Between March 2003 and March 2013, 52 PNs in patients with a solitary kidney were performed at our institution. Seven patients were excluded. Two of them were patients with tumors in transplanted kidneys, two had had prior renal surgery (Von Hippel Lindau) and three patients had undergone bench surgery and auto-transplantation. Therefore, 45 patients were available for the analysis.

Median clinical follow-up was 27.56 months (range 3 to 96), 31 patients (68%) were followed up for more than 1 year and 20 patients (44%) were followed up for more than 2 years.

### Patient characteristics

Demographic variables are summarized in Table-1. Median age was 60.4 years (r 36 to 77). Most patients were men (69%). Forty-two patients (93%) were asymptomatic at the time of diagnosis. Thirty-two were anatomical mono-renal patients (71.1%) (28 of them had undergone nephrectomies due to malignancy).

**Table 1:** Patients characteristics.

Age at surgery		
	Median (range)	60.4 years (36–77)
**Gender**		
	N° men (%)	31 (68.9)
	N° women (%)	14 (31.1)
**Symptoms**		
	No (%)	42 (93.3)
	Pain (%)	1 (2.2)
	Hematuria (%)	2 (4.4)
**Side**		
	Right (%)	25 (55.6)
	Left (%)	20 (44.4)
**Contralateral kidney**		
	Absent (%)	32 (71.1)
	Present (%)	13 (28,9)
**Body mass index**		
	Mean (SD)	30.14 (0.77)
**Comorbidities**		
	DBT* (%)	8 (17.8)
	CVD** (%)	9 (20)
	AHT*** (%)	27 (60)
	More than one (%)	17 (37)

* DBT=diabetes;

** CVD=cardiovascular disease;

*** AHT=arterial hypertension

Arterial hypertension was the most common associated comorbidity (60%) and 37% of patients had two or more comorbidities. Mean BMI was 30.14kg/m2 (SD 0.77). In 22 patients (48%) BMI was over 30kg/m^2^.

Characteristics provided by the pathology report are summarized in Table-2. Most lesions were less than 4cm (86.6%). Ninety five percent of lesions were malignant, of which the most common type was clear cell carcinoma (80%). Five patients (11.1%) had multiple lesions. Two patients (4.4%) presented with metastasis at the time of diagnosis.

**Table 2:** Pathological findings.

T		
	T1a (%)	39 (86.6)
	T1b (%)	3 (6.7)
	T2 (%)	0 (0)
	T3 (%)	3 (6.7)
**Histology type**		
	Clear cell (%)	36 (80)
	Chromophobe (%)	2 (4.4)
	Papillary (%)	4 (8.9)
	Benign (%)	3 (6.7)
**Multicentric**		
	No (%)	40 (88.9)
	Yes (%)	5 (11.1)
**Location**		
	Upper pole (%)	14 (31.1)
	Meso-renal (%)	11 (24.4)
	Lower pole (%)	20 (44.4)

### Surgical characteristics

Open surgery was the most common approach (80%). Only 13 patients (28.9%) underwent arterial clamping. Warm ischemia was applied in 11 of these patients (84.6%). Median warm ischemia time was 20.8 minutes (r 15-35). Cold ischemia time was 9 and 30 minutes in two patients, respectively.

Mean renal mass loss was 47.27mm (SD 3,45).

These findings are summarized in Table-3.

**Table 3:** Surgical features.

Approach		
	Open (%)	36 (80)
	Laparoscopic (%)	9 (20)
**Technique**		
	Enucleation (%)	9 (20)
	Polar nephrectomy (%)	32 (71.1)
	Hemi-nephrectomy (%)	4 (8.9)
**Ischemia**		
	No	32 (81.2)
	Cold	2 (4.4)
	Warm	11 (14.4)
**Loss of renal mass (mm)**		
Mean (SD*)		47.27 (3.45)

* SD=standard deviation

### Functional outcomes

Mean preoperative eGFR was 55.6mL/min/1.73m^2^ (SD 2.61). Mean eGFR 3 months after surgery was 48mL/min/1.73m^2^ (SD 3.23). Mean eGFR was-7.12mL/min (SD 2.1). This reduction was significantly different (CI 95%-11.2 to-3). The Chronic Kidney Disease (CKD) stage distribution for patients before surgery and at last follow-up is shown in Table-4. Only one patient (2.2%) had a normal renal function (eGFR>90mL/min.) before surgery and remained in that stage at last follow-up.

**Table 4:** CKD stage distribution before surgery and at last follow-up.

	Before surgery	Last follow-up
CKD Stage 1 (eGFR≥90)	1/45 (2.2%)	1/45 (2.2%)
CKD Stage 2 (eGFR 60-90)	13/45 (28.8%)	6/45 (13.3%)
CKD Stage 3 (eGFR 30-60)	30/45 (66.6%)	29/45 (64.4%)
CKD Stage 4 (eGFR 15-30)	1/45 (2.2%)	6/45 (13.3%)
CKD Stage 5 (eGFR<15 or dialisis)	0/45	3/45 (6.6%)*

* Patient who developed ESRD after 8 years had

**CKD** Stage 3 before surgery. Other 2 patients with

**ESRD** due to vascular injuries during surgery had

**CKD** Stage 2 after surgery

The results of univariate analysis are shown in Table-5. The only variables with a significant cGFR after surgery were loss of renal mass (p=0.03) and male gender (p=0.03). A reduction of 0.2mL/min/1.73m2 for each mm of tissue resected (CI 95%−0.39 to −0.02) and a reduction of 10mL/min/1.73m2 higher in male gender (CI 95%−19.1 to −0.87) were observed.

**Table 5:** Variables with impact on cGFR. Univariate analysis.

	cGFR (mL/min/m^2^)		
	Estimated (β)	CI 95%	p
Age	−0.04	0.42 to−0.5	0.85
Gender (male vs. female)*	−10.01	−19.1 to−0.87	0.03*
Absence of contralateral kidney	−0.2	10.9 to−11.4	0.96
Loss of renal mass (mm)*	−0.20	−0.39 to−0.02	0.03*
Multicentricity	−0.71	13.5 to−14.9	0.92
Ischemia (yes/no)	−1.8	7.9 to−11.8	0.78
Time of ischemia (min)	−1.5	0.2 to−3.2	0.07
BMI	0.25	1.11 to−0.61	0.55
CVD	4.8	15.9 to−6.2	0.38
AHT	1.96	11 to−7.13	0.66
DBT	6.9	18.4 to−4.52	0.22

* Significant association.

**cGFR =** variation on glomerular filtration rates.

**BMI =** body mass index. CVD: cardiovascular disease. AHT: arterial hypertension.

**DBT =** diabetes.

Both variables remained significant in the multivariate analysis with a p value of 0.004 and 0.004 respectively (CI 95%−0.4 to −0.08 and −21.4 to −4.2, respectively).

Immediate complications and their resolution are summarized in Table-6. Four patients (8.8%) experienced hemorrhage. Only one of these needed open surgery, thus achieving the preservation of the kidney. Two patients required digital angiography with supra-selective embolization of the bleeding branch and one was managed with blood transfusion. Nine patients (20%) developed a urinary fistula. Ureteral stents were placed in 3 of these patients during surgery; two patients had an unsatisfactory reconstruction of the upper urinary tract (impossibility to achieve a clean approximation of the edges) and a ureteral anastomosis was performed in one patient following an intraoperative ureteral injury. A postoperative ureteral stent was needed in 3 other patients. The remaining 3 patients solved the problem spontaneously after several days. None of these patients required open surgery. Two patients (4.4%) needed transient dialysis at the immediate postoperative period and had a full recovery of the renal function. Three patients (6.6%) developed ESRD. Two of these had vascular injuries during surgery and required dialysis at the immediate postoperative period. Both had CKD Stage 2 before surgery. The third one developed ESRD 8 years after surgery. He had CKD Stage 3 before surgery.

**Table 6:** Complications.

Complication and management	N/total (%)
Bleeding	4/45 (8.9)
Angiography	2
Open surgery	1
Transfusion	1
Urinary fistula	9/45 (20)
Ureteral stent	3
Expectant	6

### Oncologic outcomes

Four patients (8.8%) had PSMs (despite negative frozen section) and four patients (8.8%) had local recurrence (2 of these had PSMs). All patients with local recurrence had cT1 lesions (three were cT1a and one was cT1b). All of them had clear cell subtype with high Fuhrman grade (III or IV).

Five patients (11.1%) died during follow–up (all by the first year after surgery). Four patients (8.8%) died because of renal cancer and the remaining patient died because of a second tumor (gastric cancer). As mentioned above, 2 of these patients already had metastasis by the time of surgery. The first one had lung and brain metastasis and surgery was performed with cytoreductive intent. The pathology report revealed a sarcomatoid pattern in the renal lesion. The patient died six months after surgery. The second patient had suprarenal metastasis. He underwent surgery with the aim of resecting the tumor (upper pole) and the suprarenal gland. Extension to the stomach and diaphragm was found intraoperatively. Re-section was incomplete. He received sunitinib and died 8 months after surgery. The pathology report revealed clear cell subtype with Fuhrman grade IV.

The remaining 2 patients, who died because of renal cancer, had clear cell subtype Fuhrman III and papillary II subtype Fuhrman III, respectively.

Estimated 2-year overall, disease-free and cancer specific survival rates were 88.4% (CI 5% 70.5-96), 87.7% (CI 95% 68.1-96) and 92.4% (CI 95% 75-98), respectively.

## DISCUSSION

### Functional outcomes

The real impact of NSS in renal function is difficult to determine since there are many variables that can affec t the renal function during the surgery. The parameter used to establish functional outcomes also varies widely among the most important series and it is difficult to compare results. Adkins et al. and the Memorial Sloan Kettering group use the absolute change in serum creatinine (SCr) in the immediate postoperative period (5, 6). Fergany et al. used the percentage of change in SCr at the immediate postoperative period (1) in a large series of patients (n=400). However, SCr is an imperfect tool to assess renal function and identical creatinine values in the same patient at different times may not indicate equivalent renal function (7). In a cohort of 600 patients with normal SCr and normal kidneys, renal failure was detected (GFR below 60mL/min) in 25% of the patients before renal surgery (8).

Because GFR is difficult to measure in clinical practice, several formulas have been developed to estimate creatinine clearance from serum creatinine concentration, age, sex, and body size. Measuring GFR using the MDRD equation has shown the best correlation with the serum creatinine for estimating renal function (9). However, there are some limitations. Mainly, the formula was built from a CKD population. It is well-known that the relationship between GFR and creatinine is not the same in CKD and healthy subjects (10). Indeed, the MDRD formula may underestimate the true GFR in healthy subjects. There is substantial imprecision for the result of the eGFR compared to its measured value, when the measured value is≥60mL/min/1.73m^2^ (11). As mentionated, only one patient in our cohort had normal eGFR. This means that this formula is suitable for our population. La Rochelle et al. reported their results in terms of eGFR percentage decrease (7). The National Cancer institute report their results in terms of eGFR change 4 weeks and 12 months after surgery (similar to our study) (12). Additionally, several reports suggest that GFR reaches stability around 12 months after surgery. Saranchuk et al. found that GFR was stable between months 12 and 24 after surgery (6) in their cohort. Improvement in GFR was also founded by Liu et al. 12 months after surgery (12). Authors also preferred to use a late postoperative eGFR to avoid bias of many perioperative factors that may impact on renal function.

Regarding the variables that impact on functional outcomes, our cohort showed a significant association with renal mass loss and male gender. Other factors analyzed were not statistically significant. Most authors found a relationship between lower GFR and renal mass loss (measured directly or indirectly) (1, 7, 13, 14). Our results show that each mm of resected tissue determines a loss of 0.2mL/min/1.73m^2^ of GFR at 3 months following surgery. As in our study, Lane et al. also found that male gender was a negative predictor of functional outcomes of NSS (15). The effect of gender on the progression of renal disease had been established. A large meta-analysis in 2000 concluded that men with chronic renal disease of various etiologies show a more rapid decline in renal function with time than do women (16). However, they were unable to determine whether the presence of testosterone or the absence of estrogen is a determining factor. Moreover, they were unable to assess whether any nephroprotector effects of female gender are limited to premenopausal women, as would be expected if estrogen is critical.

Surprisingly, ischemia time was not a significant predictor of renal function. This is probably because we took the eGFR at 3 months after surgery and not immediately after it. A non–randomized study of 660 NSSs in patients with a solitary kidney shows that ischemia time is not an independent predictor of renal function in the long term (14). La Rochelle et al. also found that ischemia time impacted significantly only on the immediate GFR rate7. Despite this statement, we must point out that only a few patients in our cohort underwent ischemia (28.9%) and that ischemia time was very close to be significant (p=0.07). Ischemia time would have probably reached significance with a higher number of patients.

Our complication rates (urine leakage, bleeding and postoperative dialysis) seem to agree with the reported literature (1, 6, 7, 17).

Three patients developed ESRD and needed dialysis. Two of them developed ESRD immediately after surgery due to surgical vascular lesions. Delayed progression to ESRD requiring dialysis occurred in 1 patient, a fact that is consistent with previous reports (6, 7).

### Oncologic outcomes

In our series, 50% of patients with PSMs (8.8%) developed local recurrence.

Saranchuk et al. reported a local relapse of 6% and a local and distant relapse of 2% simultaneously with a mean follow-up of 32.6 months. They also reported local relapse in 13% of PSMs but did not find a clear relationship between these two factors (only 1 out of 6 patients with local relapse died due to kidney cáncer (6). La Rochelle et al. reported a local relapse of 18% and a 13% of PSMs, with a mean follow-up of 40 months (7). They found a relationship between PSMs and time to relapse. Our rates of PSMs (8.8%), local relapse (8.8%), overall mortality (11.1%) and cancer-specific mortality (8.8%) were similar to the series mentioned above.

Most series estimated their overall survival rates at 5 years, being lower in the Memorial Sloan Kettering cohort (6), which reported a 68% whereas the Mayo Clinic series17 reported a 75% of overall survival. Fergany et al. published an overall 5-year survival rate of 87% (1). The estimated 5-year cancer specific survival rates in these series were 88%, 81% and 89%, respectively.

We reported an estimated overall survival rate at 2 years of 88.4% and a cancer specific survival rate at 2 years of 92.4%. Our average follow–up is not very long (27.56 months) so we believe that a 2-year estimated survival rate is more representative for this cohort. A longer follow-up is needed, but our results appear to be similar to other series.

There are several limitations in the study such as its retrospective nature and short follow–up. Authors also are aware that the loss of renal mass is not the best parameter to evaluate loss of renal function. Not all the patients operated on are followed up at our institution so we do not have a postoperative CT scan to calculate the remaining volume of parenchyma in every patient. We believe that pathological data was the most feasible variable we had. Regarding oncologic outcomes we cannot disregard the bias of prior renal cell carcinoma in the 28 patients who were left with a solitary kidney as a result of prior nephrectomy for cancer and the impact on oncologic outcomes.

There are some strong points too. This is the first report of a Latin-American experience with patients with a single kidney. In addition, the number of patients in our cohort is not to be disregarded when compared with very important centers worldwide. Finally, we used the late GFR to assess the functional outcomes. This has proved to be a most accurate parameter to express functional aspects when compared with serum creatinine alone.

## CONCLUSIONS

NSS has acceptable results in patients with a tumor in a solitary kidney. Loss of renal mass and male gender is associated with lower postoperative GFR. Our outcomes are comparable with the published literature. Although ischemia time was not strictly statistically significant in our analysis, we believe that efforts should be made to limit renal ischemia time.
